# A Randomized Controlled Trial of Intrathecal 1% Chloroprocaine Versus 0.75% Hyperbaric Bupivacaine for Short Obstetric Procedures

**DOI:** 10.7759/cureus.97173

**Published:** 2025-11-18

**Authors:** Brandon M Togioka, Praveen Tekkali, Shangyuan Ye, Shauna K Rakshe, David Yanez, Miriam M Treggiari

**Affiliations:** 1 Anesthesiology and Perioperative Medicine, Oregon Health & Science University, Portland, USA; 2 Biostatistics, Duke University, Durham, USA; 3 Anesthesiology, Duke University, Durham, USA

**Keywords:** external cephalic version, intrathecal chloroprocaine, motor block, neuraxial anesthesia, obstetric anesthesia, postpartum tubal ligation, spinal chloroprocaine, transvaginal cerclage

## Abstract

Background

Intrathecal bupivacaine is commonly used for short obstetric procedures, including transvaginal cerclage placement, external cephalic version, and postpartum bilateral tubal ligation, despite evidence that chloroprocaine is associated with earlier postanesthesia care unit discharge after ambulatory surgery. We hypothesized that intrathecal chloroprocaine would shorten the duration of motor block after short obstetric procedures.

Methods

Single-center, randomized trial equally assigning 50 participants undergoing transvaginal cerclage placement, external cephalic version, or postpartum bilateral tubal ligation to 50 mg of 1% chloroprocaine or 10.5 mg of 0.75% hyperbaric bupivacaine. The primary endpoint was duration of motor block, defined by the count of assessments at five-minute intervals from study drug administration to knee flexion. Secondary endpoints included time to postanesthesia care unit discharge readiness, time to first ambulation, and rescue bladder catheterization. The study included an a priori halting rule to terminate if two chloroprocaine subjects failed to complete surgery under spinal anesthesia.

Results

The study was terminated at 27 subjects when a second spinal failure occurred in the chloroprocaine arm amongst patients undergoing tubal ligation surgery. Chloroprocaine was associated with decreased count of five-minute assessments until knee flexion (mean difference=7.6, 95% CI=2.9, 12.4; P=0.003), shorter time to postanesthesia care unit discharge readiness (mean difference=36.8 min, 95% CI=15.6, 58.0; P=0.001), and shorter time to first ambulation (mean difference 157 min, 95% CI=94.7, 219.3; P<0.001). Bupivacaine, relative to chloroprocaine, was associated with a 3.8-fold increased risk (95% CI=1.4, NA; P=0.02) of rescue bladder catheterization.

Conclusion

When utilized for transvaginal cerclage placement and external cephalic version, intrathecal chloroprocaine may shorten motor block, time to postanesthesia care unit discharge readiness, time to first ambulation, and reduce the frequency of bladder catheterization. Administration of 50 mg of 1% chloroprocaine intrathecally does not provide adequate anesthesia for bilateral tubal ligation surgery.

## Introduction

Transvaginal cervical cerclage placement, external cephalic version, and postpartum bilateral tubal ligation are short ambulatory procedures (<30 minutes) commonly performed during labor and delivery under spinal anesthesia. Historically, intrathecal lidocaine was used to provide short-duration spinal anesthesia for these procedures. This practice was largely abandoned due to case reports of cauda equina syndrome (mainly attributed to continuous intrathecal anesthesia) and an unacceptably high incidence of transient neurological symptoms (TNS) [1,2]. Consequently, bupivacaine, which has a low incidence of TNS and a long history of safe use in obstetric patients, became the most commonly used spinal medication for short obstetric procedures. Unfortunately, bupivacaine has a long duration of action and can prolong postanesthesia care unit (PACU) recovery [3,4]. Low-dose bupivacaine, with a shorter duration of action, has been suggested as a possible alternative; however, it is associated with inadequate anesthesia, urinary retention, and prolonged time to discharge [5,6].

Chloroprocaine, with characteristics of quick onset, short duration, and rapid postoperative recovery of motor and bladder function, is an attractive alternative. Interest in intrathecal chloroprocaine waned in the 1980s when case reports linked high-dose and sodium bisulfate-containing chloroprocaine solution to permanent neurologic injury [7,8]. In the 2000s, multiple investigations supported the safety of appropriately dosed, preservative-free intrathecal chloroprocaine [9-11]. However, there are limited trials comparing intrathecal chloroprocaine and bupivacaine in the obstetric population. Prior studies examined 3% chloroprocaine [12,13], which is formulated for epidural administration; however, only the 1% solution (Clorotekal, B. Braun Medical Inc., Melsungen, Germany) has been approved for intrathecal administration. No studies have compared intrathecal 1% chloroprocaine to bupivacaine for short obstetric procedures; therefore, the role of intrathecal 1% chloroprocaine on postoperative outcomes in the obstetric population is unknown.

We designed a randomized controlled trial with the subject, obstetric provider, investigator, and outcomes assessor blinded to treatment assignment. The primary outcome was the resolution of the motor block. Secondary outcomes were time to PACU discharge readiness, time to first ambulation, and need for rescue bladder catheterization. These outcomes were considered clinically important because prolonged PACU time increases hospital costs, earlier ambulation can reduce the risk of thromboembolic complications, and avoidance of bladder catheterization decreases the risk of urinary infection and urethral trauma, collectively improving the patient experience.

Our primary hypothesis was that intrathecal 1% chloroprocaine would shorten the time until resolution of motor block, which is typically the last criterion for PACU discharge readiness to be achieved after spinal anesthesia, compared with equivalent administration of intrathecal 0.75% hyperbaric bupivacaine. Our secondary hypotheses were that intrathecal 1% chloroprocaine would be associated with a shorter time to PACU discharge readiness and first ambulation, and a lower incidence of rescue bladder catheterization, compared to equivalent administration of intrathecal 0.75% hyperbaric bupivacaine.

## Materials and methods

Study design

The Maternal CLIMB (chloroprocaine to reduce the impact of motor block on patient recovery after short obstetric surgery) study was a single-center, randomized, controlled, parallel-group trial that compared intrathecal 1% chloroprocaine (intervention group) with 0.75% hyperbaric bupivacaine (comparison group, conventional anesthesia) in adult women. The subject, obstetric provider, investigator, and outcomes assessor were blinded to treatment assignment. The trial was registered on clinicaltrials.gov (NCT03967288) in May 2019, and Institutional Review Board approval (STUDY19846) was obtained from Oregon Health & Science University (Portland, OR, USA) in July 2019, prior to enrollment of the first subject. Written informed consent was obtained prior to subject enrollment. This manuscript contains all elements of the Consolidated Standards of Reporting Trials (CONSORT) checklist. There were no changes in study methods or surgical clinical practice after trial commencement.

Patient and public involvement

Patients and the public were not involved in study design, conduct, choice of outcome measures, or evaluation. At the time of enrollment, participants were provided with investigator contact information and an explanation of how to receive study results. 

Study population

Between October 2019 and June 2021, patients undergoing transvaginal cervical cerclage placement, external cephalic version, or postpartum bilateral tubal ligation during labor and delivery were assessed for eligibility. Eligible patients were ≥ 18 years of age, gestating a singleton pregnancy, American Society of Anesthesiologists (ASA) physical status grade II to III, and expected to receive a spinal anesthetic [14]. Exclusion criteria included study drug allergy, height < 5 feet or > 6 feet, body mass index < 18.5 kg/m^2^ or > 45 kg/m^2^, hypertransaminasemia (aspartate transaminase or alanine transaminase > twice institutional normal), renal dysfunction (estimated glomerular filtration rate < 60 ml/min/1.73 m^2^), platelet count < 80,000/microliter, international normalized ratio > 1.2, partial thromboplastin time > 36 seconds, hypotension (systolic blood pressure, SBP < 90 mmHg), lumbosacral soft tissue infection, neurologic condition representing a contraindication to spinal anesthesia (e.g. tethered spinal cord, multiple sclerosis), known atypical cholinesterase activity, and refusal of informed consent. 

Primary and secondary endpoints

The primary endpoint, duration of motor block, defined by the quantity of five-minute increments since completion of study drug administration to achievement of modified Bromage motor blockade score two [15], able to flex knees (Table 1), was actively assessed at the bedside every five minutes. An increment of five minutes between assessments was chosen to provide a balance between subject relaxation and primary endpoint precision. Knee flexion was chosen as a relevant endpoint, as it is typically the last criterion for PACU discharge readiness to be achieved after spinal anesthesia. 

**Table 1:** Modified Bromage motor blockade score used to assess decay of motor blockade after intrathecal administration of study drugs

Modified Bromage motor blockade score	Intensity of motor blockade	Physical exam finding
Score 1	No block	Free movement of legs and feet
Score 2	Partial block	Able to flex knees and move feet
Score 3	Near complete block	Unable to flex knees, but able to move feet
Score 4	Complete block	Unable to move legs or feet

Secondary endpoints were time to PACU discharge readiness, time to first ambulation, and the proportion of subjects that required rescue bladder catheterization. Patients were considered ready for PACU discharge once standard institutional criteria were met, including stable respiratory, hemodynamic, and neurologic parameters; tolerable pain and nausea; and attainment of a modified Bromage score of greater than or equal to two.

Safety endpoints

Safety endpoints were failure of spinal anesthesia requiring conversion to general anesthesia or another anesthesia technique and inadequate anesthesia (analgesic supplementation with any dose of ketamine, > 20 mg propofol, > 2 mg midazolam, or > 10 parenteral morphine equivalents). Additional safety endpoints were the cumulative dose of intraoperative phenylephrine, the proportion of subjects with intraoperative hypotension (SBP < 100 mmHg or > 20% drop from baseline), dizziness, nausea, vomiting, ephedrine administration, and anticholinergic drug administration. An independent medical monitor was tasked to review all adverse events and make a recommendation about study termination for safety.

Randomization, blinding, and recruitment

Participants were randomly allocated to 50 mg of 1% chloroprocaine or 10.5 mg of 0.75% hyperbaric bupivacaine for intrathecal administration based upon a computer-generated random sequence with a one-to-one allocation ratio without restriction. The allocation sequence was created by a member of the research division who was not involved in the study, prior to initiating recruitment. The randomization assignment was concealed in sequentially numbered opaque envelopes until the time of randomization. To maintain blinding, the outcome assessor and obstetric provider remained outside of the operating room while the anesthesia provider opened the opaque envelope, prepared, administered, and disposed of the study drug vial. 

An electronic screening tool, created within the EPIC Electronic Health Record software (Epic Systems Corporation, Verona, WI, USA), generated a weekly automated log that identified patients meeting inclusion criteria. Eligible patients were contacted by phone, and interested individuals received a follow-up email containing the consent form. Enrollment confirmation and signature of written consent were obtained in the preoperative area on the day of surgery. 

Routine patient care

All patients micturated within the five minutes preceding entry to the operating room. No subjects received bladder catheterization in the operating room. A stepwise, standard-of-care nursing protocol was used to assess and treat bladder dysfunction postoperatively (Table 2).

**Table 2:** Obstetrics and Gynecology urinary retention guideline This guideline is applicable to parturients for the eight-hour period after administration of neuraxial anesthesia or bladder catheter removal, whichever comes later

Time post-partum or post-neuraxial anesthesia	Void attempt	Unable to void or void < 150 ml	Bladder volume < 400 ml	Bladder volume ≥ 400 ml
2 hours	Greater than or equal to 150 ml: Encourage patient to void again in 2 hours.	Bladder scan	Wait for 2 hours and reassess	Intermittent bladder catheterization, reassess in 2 hours.
4 hours	Greater than or equal to 150 ml: If first void, reassess in 2 hours. If second void over 150 ml you are done measuring voids, unless indicated.	Bladder scan	Wait for 2 hours and reassess	Place indwelling bladder catheter and notify licensed independent provider.
6 hours	Greater than or equal to 150 ml and this is the second successful void. You are done measuring voids, unless indicated.	Bladder scan	Wait for 2 hours and reassess	Place indwelling bladder catheter and notify licensed independent provider.
8 hours	Greater than or equal to 150 ml and this is the second successful void. You are done measuring voids, unless indicated. If patient has not voided twice, notify licensed independent provider.	Bladder scan	Wait for 2 hours and reassess	Place indwelling bladder catheter and notify licensed independent provider.

In the sitting position, a 25-gauge pencil point spinal needle was inserted at the L3-L4 or L4-L5 intervertebral space, identified by palpation. The distal portion of the spinal needle was confirmed to be in the intrathecal space immediately prior to and after study drug administration by aspiration of cerebrospinal fluid. After the study drug administration, the patient was positioned supine. Pregnant women of 20-week gestation or later received uterine displacement with an 8.5 x 11 x 30 cm gel roll under the right ilium. A prophylactic phenylephrine infusion was initiated at 0.4 mcg/kg/min and titrated to maintain SBP within 20% of the preoperative value. Automated non-invasive blood pressure was measured at the upper arm in 2.5-minute intervals. The administration of sedative and analgesic medications, ephedrine and anticholinergic medications, intravenous crystalloid co-load, as well as the decision to convert to general anesthesia, was at the discretion of the anesthesia team and in accordance with routine practice within the study site.

Study procedures

Subjects were allocated to intrathecal administration of 50 mg (5 mL) of 1% chloroprocaine or an equipotent dose of 10.5 mg (1.4 mL) of 0.75% bupivacaine in 8.25% dextrose [16-19].The study drug was injected over two to five seconds. The subject was educated on baseline (non-blocked) sensation to blunt pinprick at the right shoulder. The most cephalad sensory level (defined as the first dermatome where sensation to pinprick was equivalent to shoulder sensation) was assessed at the midline with a blunt 18-gauge needle every one to two minutes for 15 minutes. To maintain blinding (chloroprocaine is an isobaric and bupivacaine is a hyperbaric solution), Trendelenburg positioning to facilitate cephalad spread was not allowed. 

After completion of the surgical procedure, patients were transferred to the PACU. A neurological assessment for return of motor function in the lower extremities was obtained every five minutes. Patients were queried and observed for dizziness, nausea, and vomiting. To ensure no study endpoints were missed, the blinded outcome assessor remained at the patient’s bedside until all primary and secondary endpoints were observed. A phone call and review of the electronic health record were completed seven days after hospital discharge to assess for TNS and postoperative neurologic deficits. 

Statistical analysis

Baseline characteristics collected included age, race, and ethnicity (culled from the electronic health record to align with the grant protocol), height, weight, body mass index, ASA physical status classification, hematocrit, gestational age, parity, and history of previous neuraxial anesthesia. Intraoperative characteristics collected included procedure type, surgical duration, intravenous crystalloid volume, estimated blood loss, parenteral morphine equivalents administered, most cephalad sensory level, and time from IT injection to most cephalad sensory level. Descriptive summaries included mean and standard deviation (SD) for continuous variables and frequencies (%) for categorical variables. For the primary endpoint and continuous secondary endpoints, we tested for treatment differences using the unequal variance t-test. We compared time from IT injection to Bromage score two, PACU discharge readiness, and first ambulation between study arms using Kaplan-Meier survival curves and assessed for statistical significance using the log-rank test (null hypothesis: no difference in time to event). For categorical endpoints, including the proportion of subjects that required bladder catheterization, we tested for treatment differences using Fisher’s exact test. Risk ratio confidence intervals were estimated using the bootstrap method as implemented in the R package epitools (TJ Aragon, 2020, Volume R package version 0.5-10.1; Epidemiology Tools). The study was designed to detect a difference of eight five-minute intervals from study drug administration to knee flexion (based upon retrospective institutional data) with 90% power and required a total sample of 50 patients, assuming a pooled standard deviation of 40 minutes, a two-sided hypothesis, and a 5% level of significance.

The study incorporated an a priori halting rule: it would cease if two patients in the chloroprocaine arm were unable to complete surgery under spinal anesthesia. All analyses were two-sided, and p-values < 0.05 were considered statistically significant. Analyses and generation of the fair-coin random sequence for assignment were conducted using R (version 4.4.0; R Foundation for Statistical Computing, Vienna, Austria). 

## Results

**Figure 1:**
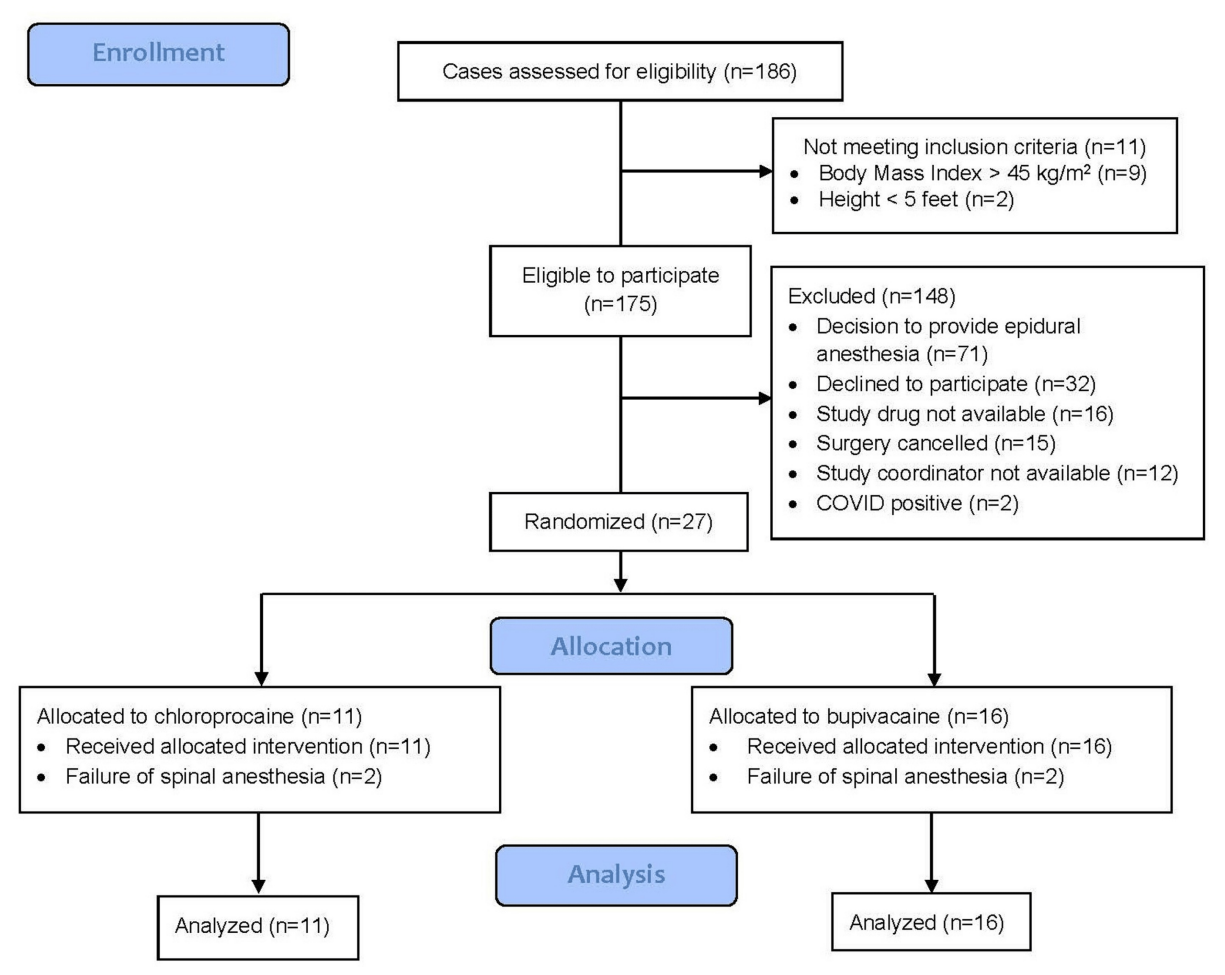
Consolidated Standards of Reporting Trials (CONSORT) diagram showing participant flow in the Maternal CLIMB study CLIMB: chloroprocaine to reduce the impact of motor block on patient recovery after short obstetric surgery

During the active study period (October 2019-June 2021), 186 cases were screened for eligibility. After chart review, 175 were deemed eligible, and 27 subjects were enrolled (Figure 1). All participants received their allocated intervention; there were no withdrawals, and complete data were obtained for all primary and secondary endpoints.

The study was halted due to the COVID-19 pandemic from March 16, 2020, to July 13, 2020. The study was halted again after three study participants required conversion to general anesthesia: two in the bupivacaine arm and one in the chloroprocaine arm on November 19, 2020. The consent form was updated to state an increased risk of spinal anesthesia failure with study participation, and the study was reinitiated on December 9, 2020. The study was permanently stopped on June 4, 2021, after a second spinal failure in the chloroprocaine arm: the fourth overall spinal failure. All failures occurred during bilateral tubal ligation surgeries.

Table 3 shows baseline population characteristics, and Table 4 shows intraoperative characteristics, stratified by treatment assignment. There was a higher representation of postpartum subjects undergoing bilateral tubal ligation in the bupivacaine (50%), in comparison to the chloroprocaine arm (27%). There were no significant differences in most cephalad sensory level achieved (P=0.77) or time to most cephalad level (8.2 min (SD=2.9) vs 8.7 min (SD=4.1); P=0.71) between chloroprocaine and bupivacaine. The most frequent cephalad levels achieved were T3-4 (45.5% of chloroprocaine and 31% of bupivacaine subjects) and T5-6 (45.5% of chloroprocaine and 50% of bupivacaine subjects). 

**Table 3:** Patient baseline characteristics stratified by randomization assignment Values represent mean (standard deviation), or count (frequency) A, Asian; ASA, American Society of Anesthesiologists; H, Hispanic; kg, kilogram; m, meter; W, White

Characteristic	Bupivacaine (n=16)	Chloroprocaine (n=11)
Age, year	30.7 (6.4)	31.7 (5.8)
Race and ethnicity (W/H/A), n (%)	9 (56%) / 6 (38%) / 1 (6%)	8 (73%) / 1 (9%) / 2 (18%)
Height, m	1.63 (0.06)	1.65 (0.06)
Weight, kg	76.2 (11.1)	80.1 (16.6)
Body mass index, kg m^-2^	28.7 (4.5)	29.5 (5.3)
ASA physical status (2/3), n (%)	16 (100%) / 0 (0%)	10 (91%) / 1 (9%)
Baseline hematocrit, %	37.0 (5.1)	34.5 (3.1)
Postpartum, n (%)	8 (50%)	3 (27%)
Gestational age of pregnant women, weeks	22.5 (10.1), n=8	27.5 (11.1), n=8
Nulliparous, n (%)	3 (19%)	4 (36%)
History of previous neuraxial anesthesia, n (%)	10 (63%)	7 (64%)

**Table 4:** Intraoperative data stratified by randomization assignment. Values represent mean (standard deviation), or count (frequency) BTL, bilateral tubal ligation; C, cerclage insertion; ECV, external cephalic version; IV, intravenous; IT, intrathecal; mg, milligram; min, minute; ml, milliliter; PME, parenteral morphine equivalent; T3-4, 3rd thoracic to 4th thoracic dermatome; T5-6, 5th thoracic to 6th thoracic dermatome; T7-11, 7th thoracic to 11th thoracic dermatome

Characteristics	Bupivacaine (n=16)	Chloroprocaine (n=11)	P-value
Surgical procedure (BTL/C/ECV), n (%)	8 (50%) / 6 (37.5%) / 2 (12.5%)	3 (27%) / 4 (36.5%) / 4 (36.5%)	0.33
Duration of surgery, min	35.9 (16.9)	31.4 (16.4)	0.49
Intraoperative IV crystalloid, ml	751 (366)	832 (526)	0.66
Estimated blood loss, ml	9 (15)	75 (202)	0.31
Intraoperative PME, mg	3.1 (6.0)	1.8 (4.1)	0.51
Most cephalad sensory level (T3-4/T5-6/T7-11), n (%)	5 (31%) / 8 (50%) / 3 (19%)	5 (45.5%) / 5 (45.5%) / 1 (9%)	0.77
Time from IT injection to most cephalad sensory level, min	8.7 (4.1)	8.2 (2.9)	0.71

Efficacy endpoints

The primary endpoint (Table 5), count of assessments every five minutes from study drug administration to achievement of Bromage score two, was lower in the chloroprocaine group compared to the bupivacaine group (16.9 (SD=4.8) vs 24.6 (SD=7.2), mean difference=7.6, 95% CI=2.9, 12.4; P=0.003). Chloroprocaine treatment was associated with shorter time from IT injection to PACU discharge readiness (93.7 min (SD=20.0) vs 130.6 min (SD=33.4), mean difference=36.8 min, 95% CI=15.6, 58.0; P=0.001) and shorter time to first ambulation (152 min (SD=58.8) vs 309 min (SD=98), mean difference 157 min, 95% CI=94.7, 219.3; P<0.001).

**Table 5:** Trial endpoints, stratified by randomization assignment Effect size is reported as estimated mean difference for continuous variables and relative risk for binary variables; values represent mean (standard deviation), or frequency IT, intrathecal; mcg, microgram; min, minute; NA, not applicable; PACU, postanesthesia care unit ^a^ NA in the context of a confidence interval represents an infinite upper or lower bound

Characteristic	Bupivacaine (n=16)	Chloroprocaine (n=11)	Mean difference or relative risk (95% CI)	P-value
Count of assessments every 5 min from IT injection to knee flexion	24.6 (7.2)	16.9 (4.8)	7.6 (2.9, 12.4)	0.003
Time from IT injection to PACU discharge readiness, min	130.6 (33.4)	93.7 (20.0)	36.8 (15.6, 58.0)	0.001
Time from IT injection to ambulation, min	309 (98.0)	152 (58.8)	157 (94.7, 219.3)	<0.001
Bladder catheterization, n (%)	11 (69%)	2 (18%)	3.8 (1.4, NA)^a^	0.02
Intraoperative hypotension, n (%)	8 (50%)	8 (73%)	0.69 (0.34, 1.3)	0.43
Intraoperative phenylephrine, mcg	478 (629)	875 (699)	-397 (-945, 152)	0.15
Dizziness, n (%)	2 (13%)	3 (27%)	0.46 (0, NA)^a^	0.37
Nausea, n (%)	2 (13%)	4 (36%)	0.34 (0, 1.4)	0.19
Vomiting, n (%)	1 (6%)	0 (0%)	NA (NA, NA)^a^	1.00
Inadequate anesthesia, n (%)	7 (44%)	3 (27%)	1.6 (0.55, NA)^a^	0.45
Spinal failure, n (%)	2 (13%)	2 (18%)	0.69 (0, NA)^a^	1.00
Ephedrine, n (%)	4 (25%)	1 (9%)	2.75 (0.46, NA)^a^	0.62
Anticholinergic, n (%)	1 (6%)	2 (18%)	0.34 (0, NA)^a^	0.55

**Figure 2:**
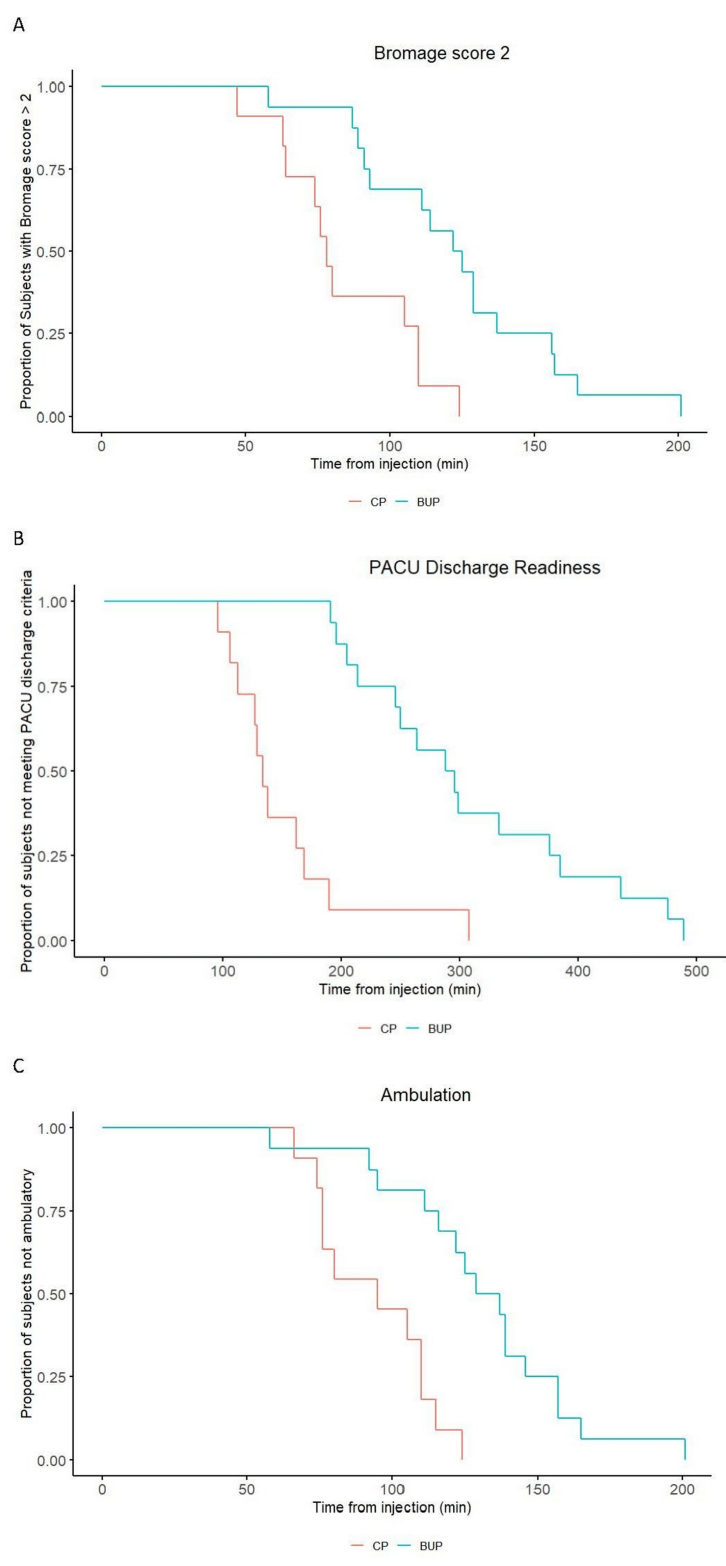
Kaplan-Meier survival curves for time from study drug injection to (A) Bromage score two, log-rank p-value <0.001, (B) PACU discharge readiness, log-rank p-value <0.001, and (C) first ambulation, log-rank p-value <0.001, in participants who received 50 mg of 1% chloroprocaine or 10.5 mg (1.4 mL) of 0.75% bupivacaine in 8.25% dextrose BUP, bupivacaine; CP, chloroprocaine; min, minutes; PACU, postanesthesia care unit

The Kaplan-Meier curves displaying time to event for (A) Bromage score two, (B) PACU discharge readiness, and (C) first ambulation, with associated log-rank p-values, are shown in Figure 2. Bupivacaine treatment was significantly associated with increased rescue bladder catheterization (RR=3.8, 95% CI=1.4, NA; P=0.02).

Safety endpoints

In an underpowered study, enrolling 54% of the target population, no difference in inadequate anesthesia (44% vs 27%, RR=1.6, 95% CI=0.55, NA) or spinal failure (13% vs 18%, RR=0.69, 95% CI=0, NA) was observed between bupivacaine and chloroprocaine.

## Discussion

A key finding of preliminary results from the Maternal CLIMB trial is that 50 mg of intrathecal 1% chloroprocaine provides inadequate anesthesia for postpartum bilateral tubal ligation. The study was terminated after two spinal failures amongst subjects receiving chloroprocaine, resulting in insufficient power to assess chloroprocaine’s clinical benefits. Our preliminary results are consistent with the known pharmacodynamics of chloroprocaine. This hypothesis-generating trial provides effect sizes for motor recovery (40 minutes), PACU discharge readiness (30 minutes), first ambulation (2.5 hours), and bladder catheterization (50%), which may inform future study design. These effect sizes, though consistent with our study hypothesis, must be interpreted cautiously due to low power.

Our primary results are better aligned with studies that include adults undergoing ambulatory procedures than studies that include pregnant women. In three small randomized trials that included subjects undergoing short ambulatory procedures, 40-50 mg chloroprocaine was observed to provide resolution of motor block 43-110 minutes faster than 7.5-10 mg of bupivacaine [16,20,21]. No difference in motor block duration was found among pregnant women receiving intrathecal chloroprocaine-1% for cesarean delivery (40 mg) or 3% for cerclage placement (50 mg)-compared to standard doses of bupivacaine (7.5 mg and 9 mg, respectively) [13,22].

We observed a 157-minute shorter time to first ambulation with chloroprocaine, which is a greater difference than that reported after short ambulatory procedures (40-148 minutes) [16,20,21]. In our study, ambulation attempts required nursing assistance, which may cause a delay between ambulation readiness and the actual time of ambulation. Differences in the approach to assessing first ambulation, along with the small sample in our study, could explain this discrepancy. Although time to ambulation was not individually assessed in studies involving pregnant subjects, one retrospective study comparing fixed dosages (7.5 mg bupivacaine vs 45 mg chloroprocaine), one pragmatic retrospective study (7 to 10 mg bupivacaine vs 40 to 60 mg chloroprocaine), and one randomized trial (9 mg bupivacaine vs 50 mg chloroprocaine) found PACU discharge readiness (of which one criterion is ambulation) to occur in the range of 73 to 152 minutes faster with chloroprocaine [12,13,23]. Consistent with previous studies [12,13,20,22], we observed more variability in time to first ambulation in the bupivacaine arm. This less predictable recovery profile has implications for PACU staffing, which accounts for most PACU costs [24].

We used a stepwise approach utilizing bladder ultrasound imaging to manage postoperative urinary retention (POUR) (Table 2). This allowed our endpoint for the assessment of POUR, rescue bladder catheterization, to be uncoupled from the time of first ambulation. In previous studies, POUR was assessed with the time of first void, and voiding trials were associated with ambulation [13,20,21]. The consistent observation is that chloroprocaine is associated with shorter duration POUR than bupivacaine [13,20,21,23]; these congruous results suggest our endpoint has convergent validity.

The high rate of failure of spinal anesthesia and inadequate anesthesia observed in our study suggest the dosages investigated were inadequate and that adjuncts, such as fentanyl, should be considered. This is especially pertinent for the population undergoing tubal ligation surgery, in whom 100% of spinal failures and 70% of cases of inadequate anesthesia occurred. Among subjects undergoing cerclage placement, the incidence of inadequate anesthesia with intrathecal bupivacaine in our cohort (17%) was aligned with previous reports (13-21%); whereas the incidence with chloroprocaine (25%) was slightly higher than the previously published range (9-20%) [12,13,23].

Strengths and limitations

Study strengths include strict blinding of the subject, obstetric provider, investigator, and outcomes assessor, with the primary outcome assessed precisely through five-minute interval questioning. Endpoints were evaluated at the bedside by a blinded assessor, with no missed assessments. While rigorous randomization, blinding, pragmatic trial design, and broad inclusion criteria enhance external validity, the generalizability of our study results is limited by low study power and a lack of precision in the results, reflected by wide confidence intervals.

The primary limitations of the Maternal CLIMB trial include its single-center design, early termination, and small sample size, which limit the strength of conclusions. Confounding was introduced by the combination of small sample size and imbalance between study arms (bupivacaine n=16, chloroprocaine n=11), resulting in unequal distribution of baseline characteristics, particularly for race, pregnancy status, and surgical procedure.

In our trial, the small sample size led to wide confidence intervals; when endpoint incidence was low in one or both arms, relative risk estimates yielded infinite or undefined upper or lower bounds. While the magnitude of the difference in some endpoints (time to motor recovery, PACU discharge readiness, time to ambulation, POUR risk) is suggestive and merits further study, the uncertainty associated with estimation from a small sample should be considered while interpreting results.

Drug doses investigated were empirically selected via institutional obstetric anesthesia faculty group consensus. The bupivacaine dose selected may be greater than the chloroprocaine dose. Previous studies in the obstetric population considered the following bupivacaine-chloroprocaine dose pairs as equipotent: 7.5 mg-40 mg, 7.5 mg-45 mg, and 9 mg-50 mg [12,13,22,23]. Though we did not propose a higher chloroprocaine dose, institutional review boards at other institutions have set a precedent of restricting evaluation of intrathecal chloroprocaine to 50 mg, and there is no safety data at higher dosages [25]. However, the incidence of inadequate anesthesia and spinal failure was similar between study arms, as was the most cephalad sensory level, suggesting the dosages were comparable.

Additional limitations include not adjusting the study drug dose for procedure-specific anesthetic requirements and not adjusting for pregnancy status. This was particularly relevant for non-pregnant subjects undergoing bilateral tubal ligation surgery, in whom all spinal failures occurred, likely due to underdosing, contributing to early study halting. Subjects undergoing postpartum tubal ligation have reduced spinal sensory and motor blockade, likely due to lower circulating progesterone levels and increased cerebrospinal fluid volume from decreased inferior vena cava compression and less epidural venous engorgement [26-28].

Significant population heterogeneity, particularly in the chloroprocaine arm, where three patients underwent tubal ligation, four patients underwent cerclage placement, and four patients underwent external cephalic version-limited the number of patients per procedure, reducing confidence in pooled estimates. Four out of 11 patients undergoing postpartum tubal ligation received general anesthesia, which may affect motor block duration, PACU discharge readiness, ambulation, and necessity for bladder catheterization, limiting the applicability of our preliminary results to patients undergoing postpartum tubal ligation surgery.

## Conclusions

The primary finding of this study pertains to patient safety. Our preliminary results suggest that intrathecal chloroprocaine, at a dosage of 50 mg, is insufficient to provide surgical anesthesia for postpartum bilateral tubal ligation surgery. Given the absence of safety data at higher dosages, dose escalation cannot be recommended. Another local anesthetic is recommended to provide spinal anesthesia for postpartum tubal ligation surgery.

The secondary contribution of this investigation lies in the effect size estimates provided for clinically relevant outcomes, including time to motor recovery, PACU discharge readiness, ambulation, and need for bladder catheterization. These estimates may serve as valuable references for future investigations, to guide design and power calculations. Low study power and population heterogeneity limit our ability to draw conclusions, highlighting the need for larger, likely multicenter, surgically homogenous studies to clarify intrathecal chloroprocaine’s role in short obstetric procedures.
